# Fibrothecoma in a Virgo Intacta Adolescent with Elevated Levels of CA 125 and B-hCG: A Case Report

**DOI:** 10.3390/children9060847

**Published:** 2022-06-08

**Authors:** Saša Raičević, Kristina Radoman, Saša Radović, Ljiljana Vučković, Filip Vukmirović

**Affiliations:** 1 Department of Gynecology and Obstetrics, Faculty of Medicine, University of Montenegro, Clinical Center of Montenegro, Podgorica 81000, Montenegro; 2 College of Health Studies, Džona Džeksona 1, Podgorica 81000, Montenegro; radoman.kristina@gmail.com; 3 Department of Paediatric Surgery, Clinical Center Montenegro, Children’s Diseases Institute, Podgorica 81000, Montenegro; sasaradovicibd@gmail.com; 4 Department of Pathology, Faculty of Medicine, University of Montenegro, Clinical Center of Montenegro, Podgorica 81000, Montenegro; ljvuckovic@gmail.com (L.V.); vuk.filip@gmail.com (F.V.)

**Keywords:** fibroma, thecoma, antigen CA 125, chorionic gonadotropin beta subunit, human, adolescent, uterine hemorrhage

## Abstract

Ovarian fibromas are benign tumors that consist of spindle cells in bundles or storiformly arranged with collagen fibers in the stroma. Thecomas resemble theca interna ovarian cells, and there is lipid material in their cytoplasm. There is an overlap in histological and immunohistochemical characteristics of these two benign tumors, and the term “fibrothecoma” was coined to describe such cases. Their incidence is extremely rare in adolescents. The subject of our study is a 15-year-old, unmarried, virgo intacta patient who was referred to us due to profuse vaginal bleeding and the loss of consciousness. A right ovary ultrasound examination exposed the formation of a hyperechoic tumor with a diameter of 41.2 mm × 29.5 mm. Findings of cancer antigen 125 (CA 125) in the amounts of 621.1 U/mL and 142.87 mIU/mL of the B-human chorionic gonadotropin (B-hCG) serum were determined. After preoperative preparation, we operated on the patient to remove a tumor with a diameter of 37 mm × 30 mm × 22 mm, smooth outer surface, solid cross-section, and yellowish white color. The diagnosis of fibrothecoma was made based on pathohistological examination. An unusual finding of fibrothecoma in a virgo intacta adolescent with profuse vaginal bleeding and increased levels of CA 125 and B-hCG may serve as a basis for broader thinking about the pathology of juvenile bleeding.

## 1. Introduction

Ovarian fibromas and fibrothecomas are rare tumors of the ovarian stroma. These tumors represent an overall of 1% to 4.7% of ovarian tumors [1]. A group of the thecoma–fibroma tumorsis encompasses a range of benign ovarian tumors, which includes fibroma, fibroma–thecoma, and thecoma, dependent on the corresponding ratio of theca cells and fibroblasts [2]. In the pediatric age range, ovarian tumors are infrequent and compose only 1–1.6% of all tumors [3]; only 1.5% of juvenile ovarian tumors are fibromas [4].

A fibroma is characterized as a benign non-hormonal tumor. On the other hand, it is acknowledged that it is estrogenic and can cause abnormal uterine bleeding [5].

Best known as the ovarian cancer marker, the cancer antigen 125 (CA 125) may also be elevated in certain benign genital and non-genital conditions [6]. Elevated CA 125 levels also occur in 29% of patients with non-gynecological cancer and some gynecological conditions of a benign character, such as pelvic inflammatory disease, endometriosis, uterine leiomyoma, and early pregnancy. Elevated levels of CA 125 even occur in patients with peritoneal, pleural, and pericardial inflammation or irritation [7]. Ovarian fibroma/fibrothecoma with elevated levels of CA 125 serum is not so common in clinical practice and can also be misdiagnosed as epithelial ovarian cancer (EOC) [7].

Elevated levels of serum B-human chorionic gonadotropin (B-hCG) are usually consistent when it comes to pregnancy or any pregnancy-related conditions, such as gestational trophoblastic tumors. Conditions other than those related to pregnancy can also show the findings of elevated B-hCG levels. Along with placental syncytiotrophoblasts, other tissues such as the testes, colon, liver, lungs, and stomach can produce B-hCG [8].

## 2. Case Report

The subject of our study is a 15-year-old unmarried, virgo intacta patient who was referred to us by a surgeon from the intensive care unit after a fall due to the loss of consciousness that resulted in a head injury (a cut on the left eyelid, which was repaired). There were no signs of acute pyramidal deficiency or subconjunctival hemorrhage. A computed tomography scan (CT) of the endocranium did not show the existence of trauma. On the first day of menstruation, the complete blood counts (SCC) were as follows: 3.75 × 10^12^/L erythrocytes (RBC); 110 g/L hemoglobin (Hgb); 0.325 L/L hematocrit (Hct); 23.09 × 10^9^/L leukocytes (WBC); and 241 × 10^9^/L platelets (PLT). These counts showed a decline in subsequent repeated determinations (2.41 × 10^12^/L RBC; 72 g/L Hgb), so two units of blood of the appropriate blood group was administered to the patient to restore levels.

**Figure 1:**
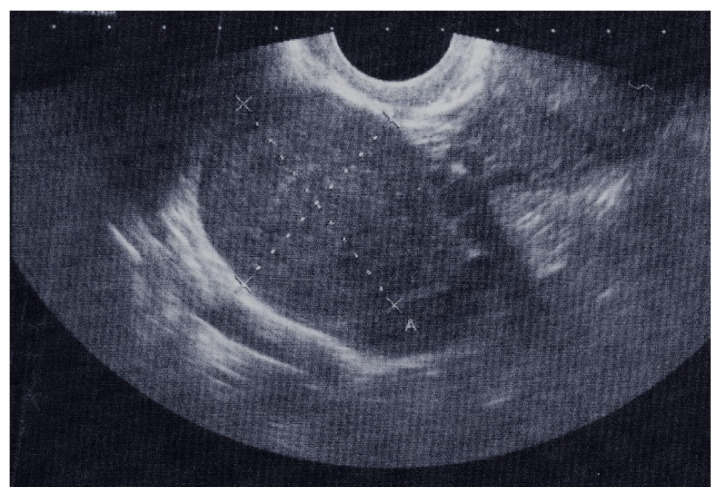
Transrectal sonography (TRS): solid tumor formation, 41.2 mm (A-X) × 29.5 mm (X-X) in diameter, with normal ovarian tissue on the periphery.

The results of a transrectal sonography (TRS) conducted by a gynecologist showed that the uterus was in anteversoflexion (AVF) of a regular shape and size. The uterine cavity was dilated up to 15 mm and filled with hyperechoic coagulum. The right hyperechoic tumor formation was 41.2 mm × 29.5 mm in diameter, with little fluid in the Douglas space, which was 21.6 mm × 8.4 mm in diameter (Figure 1). The left adnexa did not have any peculiarities. The resulting findings were 621.1 U/mL antigen CA 125 (normal 0.0–35.0) and 142.87 mIU/mL B-hCG (normal 0.0–5.0).

The patient was admitted to our clinic, where she underwent anamnestic, clinical, ultrasound, and laboratory examinations. The subject’s menarche started at the age of 12, and she has an established regular menstruation cycle that occurs on every 28 days, lasts 3–5 days, and is not painful. Her last menstruation episode started the day before the examination, with bleeding that was sparse at first but became abundant on the same day.

A rectal bimanual examination showed that the uterus was in AVF, had a normal size, and was smooth and insensitive. A solid tangerine-sized tumefaction was palpated in the area of the right adnexa. The left adnexa was easily palpated and painfully sensitive. The working diagnosis was metrorrhagia juvenilis, Tu adnexorum l. dex.

Upon admission to the gynecology clinic, the laboratory findings were as follows: 2.87 × 10^12^/L RBC; 88 g/L Hgb; 0.259 L/L Hct; 13.45 × 10^9^/L WBC; and 172 × 10^9^/L PLT. Measurements of ovarian tumor markers showed 0.8 ng/mL carcinoembryonic antigen (CEA), normal 0.0–5.0; <2.0 U/mL cancer antigen 19-9 (CA19-9), normal 0.0–37.0; 6.0 ng/mL alpha-fetoprotein (AFP), normal 0.0–8.8; and 123 U/L lactate dehydrogenase (LDH), normal 125–220.

The patient was initially administered antibiotic therapy, progesterone depo, and iron (Fe) supplementation.

As the patient clinically improved, examinations were repeatedly carried out over three days, with the following findings: 211.3 U/mL CA 125; 58.27 mIU/mL of B-hCG; 2.69 × 10^12^/L RBC; 81 g/L Hgb; 0.239 L/L Hct; 8.20 × 10^9^/L WBC; and 198 × 10^9^/L PLT. As the patient was clinically stable with only scanty bleeding, according to the law of our country, the operative therapy of adolescents was performed by a pediatric surgeon at the Institute for Children’s Diseases with the assistance of a gynecologist from our clinic. The patient was referred, with Microgynon and iron supplementation therapy, to a pediatric surgeon to plan surgery. 

Repeated findings after ten days showed 49.26 U/mL CA 125 and 11.1 mIU/mL B-hCG (Table 1), which had been almost within normal limits before the planned operation, and that the blood counts were now normal. After the examination by the pediatric surgeon, the patient was given instructions for preoperative preparation. This includes the approval of the pediatrician and anesthesiologist for the operation, as well as accompanying laboratory findings. The tumefaction was operatively removed using a laparotomy approach.

**Figure 2:**
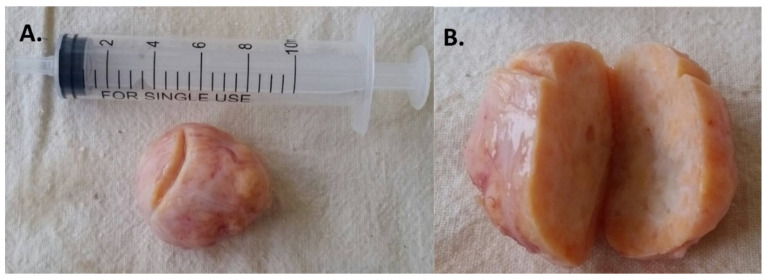
Macroscopic appearance of the removed fibrothecoma: (**A**) Oval node with smooth surface; (**B**) Yellow-gray in cross-section, solid structure.

A tumor nodule with dimensions of 37 mm × 30 mm × 22 mm and a smooth external surface, solid cross-section, and yellowish-white color (Figure 2) was submitted for pathohistological analysis. 

**Figure 3:**
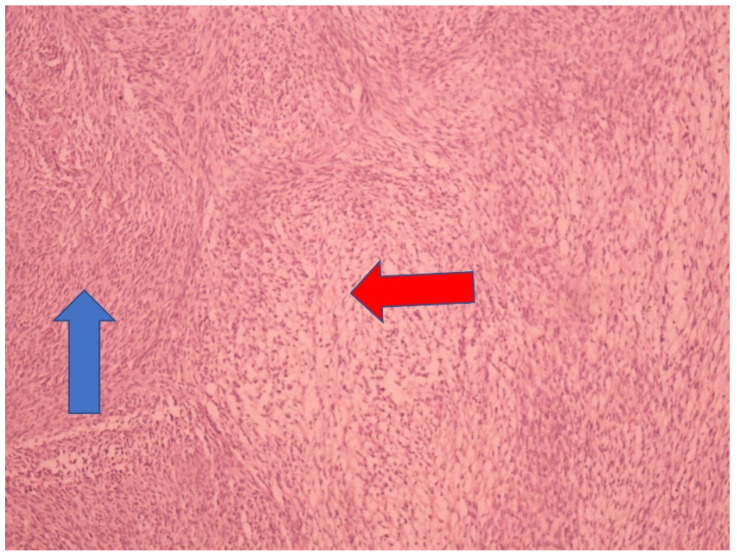
Microscopic appearance under HE staining (100×, spindle-shaped tumor cell—blue arrow, oval to round tumor cells—red arrow).

The tumor samples were formalin-fixed, paraffin-embedded, and stained with hematoxylin/eosin (HE). Immunohistochemistry (IHC) staining was performed using Vimentin (Ventana (V9) Mouse monoclonal antibody), Actin, Muscle specific (Ventana (HHF35), Mouse monoclonal antibody), and Inhibin (Ventana (alpha (R1), Mouse monoclonal antibody). A section 4 mm thick from the paraffin-embedded block was cut, and fully automatized immunoassays were performed on a Ventana BenchMark GX autostainer (Roche, Tucson, AZ, USA). The microscopic analysis revealed that the tumor nodule contains bundles of spindle-shaped, uniform cells. The cells were medium-sized, oval, and mitotically inactive. Subgroups of tumor cells were observed to have a more abundant and light cytoplasm that was spindle- to oval-shaped. Between the bundles of tumor cells, moderate amounts of collagen fibers were present (Figure 3). 

**Figure 4:**
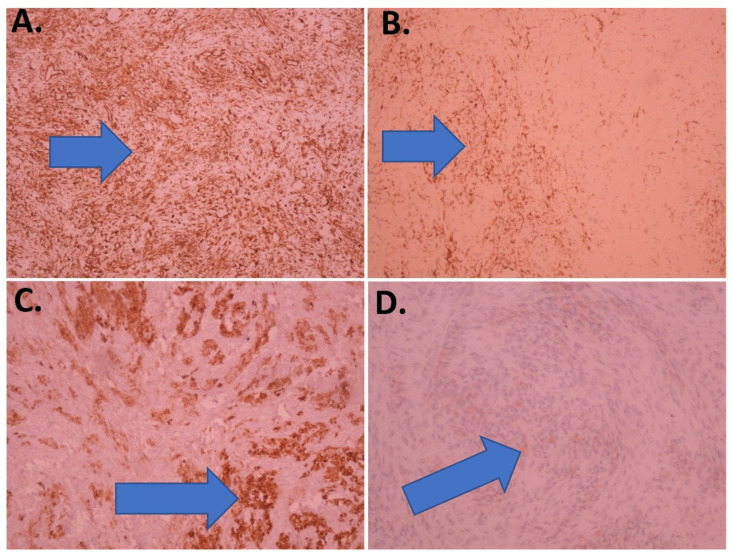
Immunohistochemical features of the fibrothecoma: (**A**) vimentin (100×); (**B**) actin (100×); (**C**) inhibin (100×); (**D**) oil red O (200×) (positive tumor cells—blue arrow).

Immunohistochemically, the spindle-shaped tumor cells were positive for vimentin and SMA (smooth muscle actin), whereas the more abundant and brighter spindle-to-coastal shaped cytoplasmic cells tested positive for vimentin and inhibin and histochemically positive for oil red O (Figure 4).

The patient was discharged after four days, and the postoperative findings were within normal limits.

Postoperatively, the examinations were repeated twice, and the findings were in order (4.45 × 10^12^/L RBC; 122 g/L Hgb; 0.378 L/L Hct; 20.5 U/mL Ca 125; <1.2 mlU/mL B-hCG.) Transabdominal sonography (TAS) showed the dimensions of the right ovary to be 24.2 × 19.3 mm, normal shape, with more clearly visible follicles.

## 3. Discussion

Ovarian tumors are rare in the pediatric population but must be considered in patients with abdominal pain and palpable masses. Although considered rare based on their incidence, they are the most common genital tumor, accounting for 60–70% of all gynecological malignancies between the ages of 10 and 19 [9]. Ovarian fibromas are benign tumors that consist of spindle cells in bundles or storiformly arranged with collagen fibers in the stroma. Thecomas resemble theca internal ovarian cells, and there is lipid material in their cytoplasm. There is a large histological and immunohistochemical overlap between the two, result-ing in the term fibrothecoma [10]. Appearance of these tumors before the age of 20 is very uncommon. However, some authors cite two peaks in frequency: the first, after the start of menopause and, the second, between the ages of 20 and 40 [11]. Zang et al. described a patient with fibrothecoma at age 14 [2], and Howel et al. described a patient 13 years of age [12].

Fibroma is characterized by a benign non-hormonal tumor [11]. On the other hand, as we have already mentioned above, it is known that fibroma is of estrogenic origin when it can cause abnormal uterine bleeding [13,14] and that it can sometimes be androgenic [15]. Functional differentiation in hormone production by luteinized theca cells or Leydig cells determines its course [15]. In rare cases, fibrothecomas can occur with endocrine manifestations related to hormonally active tumors. Meigs syndrome is the first occurrence (ovarian fibroma, hydrothorax, and ascites), observed in 1–10% of ovarian fibromas. After ablating the tumor, hydrothorax and ascites regress rapidly. Another association is named Gorlin–Goltz syndrome, i.e., the basal nevus syndrome. Ultimately, ovarian fibromas may also be linked with familial polyposis such as Gardner’s and Richard’s syndromes and Peutz–Jeghers syndrome [11].

Normal levels of CA 125 serum are <35.0 U/mL. Unusually, in our case report of fibrothecoma, the CA 125 (621.1 U/mL) level was increased. Using CA 125 serum as an ovarian cancer tumor marker has been proposed as a valuable tool to help distinguish between benign and malignant neoplasms [16]. However, the precision of CA 125 is constricted [17]. Chechia et al. [3] measured the levels of CA 125 serum in 21 patients with fibroma/fibrothecoma and found that 14.2% had values of >35 U/mL [11]. Chen et al. found that 12 out of 58 (20.6%) patients with ovarian fibromas/fibrothecoma had a serum CA 125 value of >35 U/mL [18].

The CA 125 serum levels in pregnant women are known to increase to a certain degree in the first trimester of pregnancy and then to start descending during the second and third trimesters to the scope found in non-pregnant women [19]. The maximum value of 550 IU/mL is recorded in the first trimester [20]. Adeku et al. found that the mean level of CA 125 serum in women who aborted was 34.8 ± 1.4 IU/mL, whereas the mean level of CA 125 serum in women whose pregnancies continued until their term (non-aborted) was 27.3 ± 1.2 IU/mL [6]. Amampai et al. [21] concluded that for normally pregnant women, the level of CA 125 serum was largely within the normaly allowed limits. Only 10% were found to have an increase in CA 125. Therefore, an increase in CA 125 serum in pregnant women with ovarian tumors should be of concern as a possible cause of ovarian tumors, in addition to pregnancy [21].

Normal levels of B-hCG serum are 0.0–5.0 mIU/mL. In our case report of fibrothecoma, the B-hCG level (142.87 mIU/mL) was increased in a 15-year-old patient, virgo intacta, coming from a conservative family. Nigam et al. described a case of distorted ovarian fibroma with elevated B-hCG levels and ascites that mimic an ectopic ruptured pregnancy in a young married woman who had not given birth. Elevated levels of B-hCG in this case were attributed to chemical pregnancy [22]. 

Measurable levels of B-hCG serum is generally consistent with pregnancy or pregnancy-related conditions, such as gestational trophoblastic tumors. B-hCG can also be discovered in non-pregnant conditions [8]. If elevated levels of B-hCG serum are found in the presence of an ovarian mass, there is no evident intrauterine gestational sac, and ectopic pregnancy is ruled out, cases of B-hCG-secreting tumors including dysgerminomas, polyembryoma, placental site trophoblastic tumors, or choriocarcinoma must be considered [23]. Cases of mixed polyembrioma and immature teratoma resulting in elevated hCG serum and alpha-fetoprotein, or cases of torqued dermoid cyst mimicking ectopic rupture have been reported [24]. High levels of B-hCG serum can instigate paraneoplastic syndrome with symptoms such as amenorrhea, irregular vaginal bleeding, or hyperemesis; these symptoms may be misunderstood as indicators of intrauterine or ectopic pregnancy [8]. Olsen et al. described five reasons for a positive B-hCG result other than pregnancy: false-positive hCG or “phantom hCG”, pituitary hCG, the exogenous use of hCG, trophoblastic neoplasms, and nontrophoblastic neoplasms [25].

In our case, we cannot give a clear explanation for the drop in Ca 125 and HCG levels without any treatment. We hypothesize that Ca 125 spikes may be due to mechanical irritation of the peritoneum or pelvic inflammatory disease and that B-hCG spikes may be due to false-positive hCG or “phantom hCG”. We were considering early pregnancy and possible abortion. However, we obtained information from the anamnesis that the patient was virgo intacta. This was confirmed by a clinical bimanual rectal examination, where the intact hymen was clearly visible. We also thought about possible petting as a possibility of becoming pregnant, but since the presence of the patient’s mother is mandatory, we gave up on further anamnestic examination.

Sonography is predominantly used as a first choice imaging technique to assess pathological ovarian abnormalities. In the case of transabdominal sonography (TAS) giving a blurred picture and transvaginal sonography (TVS) not being able to be used or having relative contraindications, as in the cases of virgo intacta, agenesis of the vagina, and remature rupture of the membranes, we used a transrectal sonography (TRS). In a study by Timor-Tritsch et al., in which a TAS and TRS results of 42 patients have been compared, TRS indication for 23 patients was a virgo intacta diagnosis [26]. Regardless, the ultrasound characteristics of fibromas and fibrothecomas are usually nonspecific [27] and magnetic resonance imaging is often required to further differentiate ovarian fibromas and fibrothecomas from other solid ovarian masses, especially leiomyomas with pedunculated or broad ligaments. Imaging is important for distinguishing fibromas and fibrothecomas from malignant ovarian tumors, especially when they are present as a solid ovarian mass associated with ascites and pleural effusion. Nevertheless, many studies have been evaluating the ultrasound characteristics of fibromas and fibrothecomas, which are often general (i.e., non-specific) [27], and when MRI characteristics were studied, studies either had small sample sizes or were focused on specific characteristics of fibromas and fibrothecomas. Even up to this day, radiologists often face difficulties in accurately diagnosing fibromas and fibrothecomas, especially their distinction from fibromas [28]. On the basis of the CT findings in 24 cases of thecoma–fibroma and related reports, Zang et al. [2] summarized the CT characteristics: most tumors occurred unilaterally with clear borders, and round or oval shapes, accounting for 84.62% (22/26) of the study; the tumor content was mostly solid; smaller tumors showed a homogeneous density, while the larger tumors were often heterogeneous; and low-density areas such as cysts or patches could be found within them [2].

Surgery is the advisable treatment for ovarian fibromas, and conservative treatment should be used with caution. As with other ovarian tumors, surgery is the treatment of personal choice for solid tumors. Conservative surgery with unilateral salpingo-oophorectomy or the excision of the ovarian mass is recommended for patients of reproductive age, whereas more radical surgeries should be considered in older patients. In the literature, laparotomy has been used for removal of ovarian fibromas in most cases, and laparoscopic access has rarely been reported. Leung et al. performed laparotomy in 78.3% of cases and laparoscopic surgery in 21.7% of cases [1]. Another option is a minimal access surgery, particularly when tumor is moderate to small in size [1]. In our case, we opted for laparotomy and the removal of the ovarian tumor mass.

Laparoscopic surgery has many advantages in terms of result, hospital stay, postoperative pain, better cosmetic result, better visualization of the ovary during surgery, etc. The laparoscopic surgery in this particular case could not be performed because the child has congenital persistent pulmonary hypertension. The insufflation of CO_2_ would compromise the respiratory pattern.

Of course, a definitive diagnosis is obtained microscopically, and histological findings generally allow for a diagnosis to be made without difficulty. However, in some cases, differential diagnosis is more difficult for thecomas, especially fibrotic thecomas, stromal tumors, fibromatosis, and massive ovarian edemas [28]. Diagnoses of these groups of tumors (fibroma/thecoma group) are, in most cases, performed by analyzing their morphology on HE-prepared slides. Bearing in mind the fact that these ovarian tumors are sex cord-stromal tumors of mesenchymal origin, these tumors are typically diffusely positive to vimentin. Fibromas are frequently positive for smooth muscle actin, and rarely positive for CD34 and desmin. In some cases, inhibin and calretinin may be focally and weakly positive. Thecomas, in addition to being positive to vimentin, are also positive for inhibin, calretinin, and CD10 [7,29]. Despite the fact that the diagnoses of these groups of tumors are in most cases performed on HE-prepared slides, we performed immunohistochemistry in order to confirm the fibroma and thecoma components of the tumor. In the immunohistochemistry protocol, a finding that proved helpful in finalizing the diagnosis was the strong vimentin staining, which is characteristic of ovarian fibrothecomas [30]. The source of serum CA 125 in ovarian fibroma/fibrothecoma remains unclear. Some previous studies have considered that elevated serum CA 125 originates from nontumor cells and that the same biochemical factors may be involved in this condition, such as a mechanical irritation of peritoneum or an increase in intraperitoneal pressure form a large volume of tumor and ascites [7].

## 4. Conclusions

The incidence of fibrothecomas in adolescents is extremely rare. This case report involes the investigation of a virgo intacta adolescent patient with profuse vaginal bleeding and increased levels of CA 125 and B-hCG. This case report can serve as a basis for broader thinking about the pathology of juvenile bleeding.

## Figures and Tables

**Table 1:** CA 125 and B-hCG findings in the patient.

	CA 125 U/mL (0.0–35.0)	B-hCG mlU/mL (0.0–5.0)
At the first examination	621.1	142.87
After three days	211.3	58.27
After two weeks	49.26	11.1
Before surgery (after two months)	39.4	1.05
After surgery	20.5	<1.2
